# Risk Factor Assessment and a Ten-Year Experience of DDH Screening in a Well-Child Population

**DOI:** 10.1155/2019/7213681

**Published:** 2019-08-04

**Authors:** Bahar Kural, Esra Devecioğlu Karapınar, Pınar Yılmazbaş, Tijen Eren, Gülbin Gökçay

**Affiliations:** ^1^MD, Ph.D. Pediatrician, Istanbul University Institute of Child Health, Department of Social Pediatrics, Istanbul, Turkey; ^2^MD, Pediatrician, Istanbul University Institute of Child Health, Department of Social Pediatrics, Istanbul, Turkey; ^3^MD, Ph.D., Family Medicine Specialist, Istanbul University Institute of Child Health, Department of Social Pediatrics, Istanbul, Turkey; ^4^Professor of Pediatrics, Istanbul University Institute of Child Health, Department of Social Pediatrics, Istanbul, Turkey

## Abstract

**Aim:**

Risk based screening for developmental dysplasia of the hip (DDH) with ultrasound is common. However, risk factors vary from one country to the other since data are insufficient to give clear recommendations. We aimed to evaluate the risk factors for developmental dysplasia of the hip (DDH).

**Methods:**

In this retrospective case-control study, the health records of all children, who were followed up between 2004 and 2014 at a well-child unit, were investigated for the diagnosis of DDH in Turkey. Of 9758 children, 57 children were found to have abnormal ultrasonographic findings (according to Graf classification) and these constituted the case group. As the control group, healthy 228 children who matched the case children in birth months were selected. Two groups were compared for the risk factors.

**Results:**

A total of 19516 hips of 9758 children were examined for DDH. 97 hips of 57 children were found to have abnormal ultrasonographic findings. When the two groups were compared, breech presentation, multiple pregnancy, and torticollis were identified as risk factors. The female sex was also found to have a significantly high prevalence among the children in the case group. Limited hip abduction, positive Ortolani, and Barlow signs were important clinical findings in the case group.

**Conclusion:**

According to our findings, breech presentation, female sex, torticollis, and multiple pregnancy were found to be the risk factors of this disorder. Infants with these risk factors should be investigated carefully for DDH.

## 1. Introduction

Screening programs for DDH have been present for many years. Different programs include pure clinical examination, selective ultrasonographic screening of at-risk newborns or universal neonatal ultrasonographic screening [1]. Age appropriate imaging is recommended by the American Academy of Pediatrics for female infants born breech or with family history of DDH [2]. In the United Kingdom, clinical hip instability in physical examination, family history of first degree relative requiring DDH treatment, breech position, multiple births if any of the babies is breech presentation are seen as risks requiring ultrasonographic investigation [3]. The screening of all newborns at birth for DDH using ultrasound imaging is standard practice in Germany, Austria, and Switzerland [4].

Risk based screening program was started in Turkey in 2013 as a pilot study and countrywide in 2014. All neonates with family history of DDH up to third-degree relatives, oligo/hydoamnios, breech presentation, foot deformities, plagiocephaly, scoliosis, congenital muscular torticollis (CMT), pelvic obliquity, adduction contracture of the hip, multiple pregnancy, and firstborn girl of the family are referred for ultrasound scanning [5].

Risk based screening for developmental dysplasia of the hip (DDH) with ultrasound is common in the world. On the other hand, risk factors vary from one country to the other since data are insufficient to give clear recommendations [6, 7].

In this retrospective case-control study, we aimed to investigate the risk factors for DDH in a well-child unit in Turkey.

## 2. Methods

**Figure 1:**
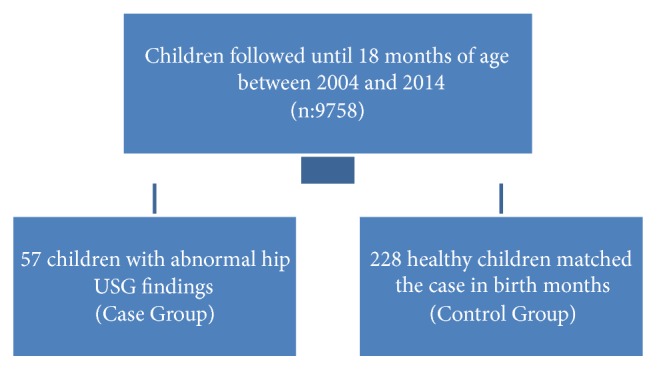
The study design.

This retrospectively designed case-control study was carried out in the Well-Child Unit of Istanbul University Hospital in Turkey. Each child had a health record starting from 1 month of age at the unit. The health records of all children, who were followed between January 2004 and December 2014 were investigated for the diagnosis of DDH. Children admitted to the Unit include term infants born at the maternity clinic of the same hospital. Children were followed every 2 months for the first 6 months and every 3 months until 18 months of age and every 6 months thereafter until 10 years of age according to the program of Well-Child Unit. At each visit, detailed physical examination was carried out and findings were recorded in the personal files of the children. The basic sociodemographic information of each child was also kept. Physical examination and risk assessment for DDH were conducted for each infant starting at 1 month of age in the Unit. Barlow and Ortolani test were performed for this purpose until the third month of life. Asymmetric thigh or perineal crease, an apparent short leg (positive Galeazzi sign) and limitation of hip abduction were also sought as clinical findings. Children with risk factors listed in Table 1 underwent ultrasound examination at the unit. The walking pattern of the child was also evaluated during the visits. Detailed family, perinatal and natal history (including birth weight) were taken for each child at the first admission. According to the unit's policy, parents were guided about the clothing for the children and swaddling was not recommended. Sonographic examinations and classifications were performed in combination with the Graf technique [8]. The final combined clinical and sonographical examination for each child was carried out by a pediatrician and/or a pediatric resident under supervision of the senior author (GG) before the referral of the children to the orthopedic surgeon. Infants older than 3 months with abnormal ultrasonographic findings (Graf classification type IIa, IIc, and D,III/IV) constituted the case group[8]. Two children before and two after each case child as listed in the records, who were born in the same month and in the same year were selected as controls; therefore four controls were selected per case. The control group encompassed 228 children. The study design was given in Figure 1. All children in the study had been followed up until 18 months of age.

Ethical approval and necessary institutional permissions were obtained for the study.

## 3. Statistical Analyses

The NCSS (Number Cruncher Statistical System) 2007 (Kaysville, UT, USA) program was used for the statistical analysis. Student's t test, Pearson *χ*^2^ test, and Fisher's exact test were carried out for the analysis. Results were evaluated at the 95% confidence interval (95% CI) range and p<0.05 significance level.

## 4. Results

Of the 9758 children, 57 (0.58%) had abnormal ultrasonographic findings. The age distribution of the cases at the first ultrasonography is given in Table 2. This study encompassed the sonographic findings of 97 hips in 57 infants.

The orthopedic treatment and follow-up data of 57 children were evaluated. The distribution of clinical findings and risk factors in case and control groups are presented in Table 3. In the case group, one child with arthrogryposis multiplex had operation. An abduction brace such as a Pavlik harness was applied to 19 infants. As a result of this finding, true DDH was 0.2%. The distribution of the risk factors in case group according to management protocol are provided in Table 4. Some children have more than one risk factor. The distribution of risk factors was similar. The 37 untreated infants were carefully followed clinically and sonographically. Repeated ultrasonographical and radiological examination of these infants were normal by the age of 18 months.

Results of unilateral analysis of risk factors which were statistically significant are given in Table 5. Our results showed a statistically significant difference in the proportion of females versus males, who were at risk for developing DDH; female children had 2.27 times greater odds of DDH risk than male children. Cases had three times greater odds of breech presentation and eight times greater odds of CMT and four times greater odds of multiple pregnancy than children in the control group.

Limited hip abduction was significantly higher in the case group than in the control group. Unilateral limitation of abduction had a positive predictive value of 82%. The Galeazzi sign was not noted in both groups.

All children were followed-up until the walking age and no late case was identified.

## 5. Discussion

A 10-year DDH risk-based screening experience was presented in this study. Breech presentation, female sex, torticollis, and multiple pregnancy were found to be the statistically significant risk factors. The significant findings were the Ortolani and Barlow maneuvers and limitation of hip abduction.

The early identification of children with DDH is valuable as it allows for less invasive corrective procedures than if DDH is identified late [9]. In our study, the oldest age for diagnosis of DDH was 4 months.

Peled et al. defined “true DDH” as a hip with a subsequent treatment and that definition lowered the incidence to 0.5% [10]. According to this definition, we had only 20 (0.2%) children with treatment (surgery or brace). Studies from Turkey have revealed a wide range of 0.5-28.1% depending on screening method, definition and population [11, 12].

Lipton et al. investigated hips that showed a positive Ortolani sign on ultrasound and concluded that this sign indicated an abnormal ultrasonographic finding [13]. Choudry et al. suggested that limitation of hip abduction should be actively sought after 8 weeks of age and if present, ultrasonographic or radiographic examination should be performed [14]. Roposch et al. showed that even among pediatric orthopedic surgeons, there were wide variations in the diagnostic criteria for DDH in infants [15]. In our study, all children with positive Ortolani and Barlow maneuvers were in the case group. Unilateral limitation of abduction had a positive predictive value of 82% in the group.

In a Dutch study, total of 683 babies between 3 to 10 months old, limitation of abduction had a positive predictive value of 43% [16]. Our findings revealed that in the unit, clinical screening of DDH was successfully carried out by the physicians.

In our study, a family history of DDH was not found to be a risk factor. This may be because of our limited definition of “family history”. The definition of family history in the literature ranges from unspecified hip disorders to hip dislocation and from first-degree relatives (parents and siblings) to any relative (even if distant or vague) with hip problems or DDH [17]. As we evaluated the health records retrospectively, we could not extend the definition of family history. Recent studies suggested that history of DDH and hip osteoarthritis among any family members should be considered as risk factor [18].

Female sex -whether in a first-born or not-is a well- known risk factor for DDH, probably because of increased ligamentous laxity due to the circulating maternal hormone relaxin [19]. In our study, the risk in females was identified to be 2.27 times higher than that in males. In a meta-analysis of 31 studies, the relative ratio (RR) in newborn females was found to be 2.54 (95% CI: 2.11-3.05) times more prevalent than in males [20].

The definition of oligohydramnios has changed over time with the use of ultrasonography [21, 22]. Therefore, there are conflicting results in the literature about oligohydramnios as a risk factor for DDH[22]. In a study, Paton stated that oligohydramnios did not appear to be true risk factor in the development of pathological DDH [23]. In our study, it was also not found to be a risk factor for DDH.

Findings about multiple pregnancy as a risk factor for DDH are also controversial. Some authors did not recommend routine ultrasound screening for twins and triplets [24–26]. In our study, multiple pregnancy (twins or triplets) was found to be a risk factor for DDH. This may be due to the additional risk factors of multiple pregnancy[24].

The frequency of DDH among children with breech presentation was reported to be between 17-23% [27]. It has been considered the most important environmental factor for DDH [22]. In our study, breech presentation was confirmed as a risk factor and 36% of all children (cases and controls) with breech had DDH. The risk of DDH is the highest in frank breech presentation (one or both knees extended) [28]. In our study, breech history was obtained from patients' files and there was no detailed types of breech presentation.

There is no consensus on the routine hip imaging screening of patients with congenital muscular torticollis [17, 29, 30]. In our study, torticollis seemed to be a risk factor for DDH. All CMT cases with DDH received brace treatment. Our finding led us to think that an ultrasonographic evaluation of the hips should be carried out for children with CMT.

Our study had some limitations. First, as we mentioned above, our definition of family history was limited in this study. Second, preterm infants were not included. Finally, we did not gather information about swaddling. Further prospective studies that investigate these factors are needed to confirm our findings.

## 6. Conclusion

Our study had important findings about the risk factors for DDH. The results showed that physical examination is still an important tool in the screening. Breech presentation, female sex, torticollis, and multiple pregnancy seemed to be risk factors for this disorder and infants at risk should be investigated carefully by means of ultrasonography.

## Figures and Tables

**Table 1:** Risk factors used for the screening of developmental dysplasia of the hip in the unit.

Family history (first-degree relatives)

Multiple pregnancy

Breech presentation

Oligohydramnios

Congenital muscular torticollis

Foot deformities

Pathological clinical findings(Positive Ortolani and Barlow maneuvers, Positive Galeazzi sign, limited abduction, and asymmetric crease)

**Table 2:** Age distribution of the cases at the first hip ultrasonography (USG).

Timing of USG (weeks)	n(%)
4	24 (42,1)

6	5 (8,8)

8	26 (45,6)

12	1 (1,8)

16	1 (1,8)

Total	57 (100)

**Table 3:** Distribution of risk factors and clinical findings in the case and control groups (n= 285).

	*Case n:57 *n (%)	*Control n:228 *n (%)	*p value*
Family history of DDH	5 (8.8)	11 (4.8)	^*b*^ *0.329*
Female sex	40 (70,2)	116 (50,9)	^*b*^ *0,009*
Breech delivery	9 (15.8)	16 (7.0)	^*a*^ *0.036*
Multiple pregnancy	5 (8.8)	5 (2.2)	^*b*^ *0.030*
Oligohydramnios	1 (1.8)	8 (3.5)	^*b*^ *0.693*

Ortolani maneuver positivity	4 (7.0)	0	*0.001*

Barlow maneuver positivity	3 (5.3)	0	*0.008*

Asymmetric thigh or perineal crease	14 (24.6)	58 (25.4)	^*a*^ *0.892*

Limited hip abduction	27 (47.4)	6 (2.6)	*0.001*

Congenital muscular torticollis	3 (5.3)	1 (0.4)	^*b*^ *0.026*

Foot deformities	2 (3.5)	2 (0.9)	*0.180*

^*a*^ *Pearson χ* ^2^ *test.* ^*b*^ *Fisher's exact test.*

**Table 4:** Distribution of risk factors in case group according to management.

Risk factors	Brace treatment	Follow-up without treatment *(n:37)*	Operation
	*(n:19)*		*(n:1)∗*
Family history of DDH	2	3	-

Female sex	15	24	1

Breech delivery	4	5	-

Multiple pregnancy	1	4	-

Oligohydramnios	-	1	-

Ortolani maneuver positivity	1	3	-

Barlow maneuver positivity	1	2	-

Asymmetric thigh or perineal crease	-	14	-

Limited hip abduction	18	8	1

Congenital muscular torticollis	3	-	-

Foot deformities	2	-	-

*∗*: the patient had arthrogryposis multiplex.

**Table 5:** Results of unilateral analysis of risk factors.

Risk Factor	*Odds Ratio (OR)*	*Confidence Interval (CI)*
Female sex	2.27	1.21-4.24

Breech delivery	2.96	1.25-7.04

Multiple pregnancy	3.83	1.17-12.53

Congenital muscular torticollis	7.88	1.28-48.33

Limited hip abduction	33.3	12.7-87.25

## Data Availability

The data used to support the findings of this study are available from the corresponding author upon request.
